# Sphenoidal Foramen Ovale in the Slovenian Population: An Anatomical Evaluation with Clinical Correlations

**DOI:** 10.3390/diagnostics13050962

**Published:** 2023-03-03

**Authors:** Žiga Šink, Nejc Umek, Armin Alibegović, Erika Cvetko

**Affiliations:** 1 Institute of Anatomy, Faculty of Medicine, University of Ljubljana, Korytkova 2, 1000 Ljubljana, Slovenia; 2 Institute of Forensic Medicine, Faculty of Medicine, University of Ljubljana, Korytkova 2, 1000 Ljubljana, Slovenia

**Keywords:** foramen ovale, sphenoid bone, anatomical variations, morphometry, trigeminal nerve

## Abstract

The foramen ovale (FO) is a crucial feature of the skull base, serving as a passage for clinically important neurovascular structures. The present study aimed to provide a comprehensive morphometric and morphologic analysis of the FO and highlight the clinical significance of the anatomical characterization. A total of 267 FO were analyzed in skulls obtained from deceased inhabitants of the Slovenian territory. The anteroposterior (length) and the transverse (width) diameters were measured using a digital sliding vernier caliper. Dimensions, shape, and anatomical variations of FO were analyzed. The mean length and width of the FO were 7.13 and 3.71 mm on the right side and 7.20 and 3.88 mm on the left side. The most frequently observed shape was oval (37.1%), followed by almond (28.1%), irregular (21.0%), D-shaped (4.5%), round (3.0%), pear-shaped (1.9%), kidney-shaped (1.5%), elongated (1.5%), triangular (0.7%), and slit-like (0.7%). In addition, marginal outgrowths (16.6%) and several anatomical variations were noted, including duplications, confluences, and obstruction due to a complete (5.6%) or incomplete (8.2%) pterygospinous bar. Our observations revealed substantial interindividual variation in the anatomical characteristics of the FO in the studied population, which could potentially impact the feasibility and safety of neurosurgical diagnostic and therapeutic procedures.

## 1. Introduction

The sphenoid bone constitutes the base of the skull between the frontal, temporal, and occipital bones. Its greater wing contains three consistent and a few small variable foramina. The consistent foramina are the foramen ovale (FO), the foramen rotundum (FR), and the foramen spinosum (FS). They act as conduits for several cranial neurovascular structures and are important in various clinical conditions and procedures.

The FO is located in the posterior aspect of the greater wing of the sphenoid bone, posterolateral to the FR, anteromedial to the FS, and lateral to the foramen lacerum (FL). It connects the middle cranial fossa to the infratemporal fossa and transmits the mandibular nerve, the lesser petrosal nerve, the accessory meningeal artery, the emissary veins, and the anterior trunk of the middle meningeal sinus [1,2]. Its location renders it useful in various diagnostic and therapeutic procedures, including administering anesthesia to the mandibular nerve, trigeminal rhizotomy for managing trigeminal neuralgia, percutaneous biopsy of parasellar lesions, and electroencephalographic temporal lobe analysis during selective amygdalohippocampectomy [1,3,4,5,6].

Accordingly, the structural characteristics of the FO bear remarkable clinical significance. Anatomical variations of the FO are a commonly observed phenomenon that may interfere with transoval cannulation and hinder surgical access to this area. In addition, aberrant FO anatomy is also etiologically associated with certain pathologies. For example, compression of the mandibular nerve in this region from anomalous shapes or bony outgrowths may lead to the development of trigeminal neuralgia [7]. This study aimed to determine and compare the morphometric and morphological features of the FO in adult human skulls from the Slovenian population with those previously reported in the literature and highlight potential clinical relevance.

## 2. Materials and Methods

The analysis was performed on 126 whole dried adult human skulls and an additional 15 dried human skull halves (3 right and 12 left) of undetermined sex and age, obtained from bodies donated by inhabitants from the territory of the Republic of Slovenia between the years 1965 and 2020 to the anatomical collection of the Institute of Anatomy of the Faculty of Medicine of the University of Ljubljana. Additionally, 30 whole dried adult human skulls were analyzed from the bone collection of the Institute of Forensic Medicine, Faculty of Medicine, University of Ljubljana, Slovenia. A total of 267 FO were analyzed in all specimens. Skulls with evidence of physical damage to the structures of interest, confirmed by inspection with magnifying lenses, were excluded from the analyses.

The greater wings of sphenoid bones were observed from the extracranial and intracranial views of the skull base for visualization and measurement of the FO. A thin wire was used to confirm the patency of foramina and rule out false passages. The FO was measured along the anteroposterior (length) and transverse (width) diameters using a digital sliding vernier caliper with a precision of 0.01 mm. The distance between the FO and FS was measured using the same method. The shape of the FO and its potential anatomical variations (marginal bony outgrowths, divisions, duplications, confluences) were carefully recorded and photographed. Additionally, the FO was classified as either foramen-like or canal-like. FO was defined as canal-like when the distance between its outer and inner margins exceeded 2 mm. To minimize the measurement error and bias, each morphometric and morphological parameter was independently measured or assessed twice by at least two independent researchers, and the mean value was used for the analysis. Discordant descriptions or measurements were further evaluated by the other two authors, and consensus was reached through a joint discussion among all authors. Previous studies were also referenced to standardize evaluation protocols and anatomical descriptions [8,9,10,11,12,13,14,15].

Statistical analysis was performed using GraphPad Prism 9 (GraphPad Software, San Diego, CA, USA). Data are presented as means (standard deviation) or frequencies (proportion). Differences between the right and left sides were analyzed using a paired sample t-test. The differences were considered statistically significant at *p* < 0.05. The Kolmogorov–Smirnov test was performed for the evaluation of the normality of the distributions. A nonparametric χ2 test was used to detect differences between proportions. The obtained data were compared with previous reports.

## 3. Results

The FO was present in all analyzed 267 sides of dried adult human skulls. The mean anteroposterior diameter or length (longest axis) of the FO was 7.13 mm on the right side and 7.20 mm on the left side. The mean transverse diameter or width (shortest axis) of the FO was 3.71 mm on the right side and 3.88 mm on the left side. The morphometric features of the FO are summarized in Table 1. No statistically significant differences were found in any measured parameter between the left and right sides.

**Figure 1:**
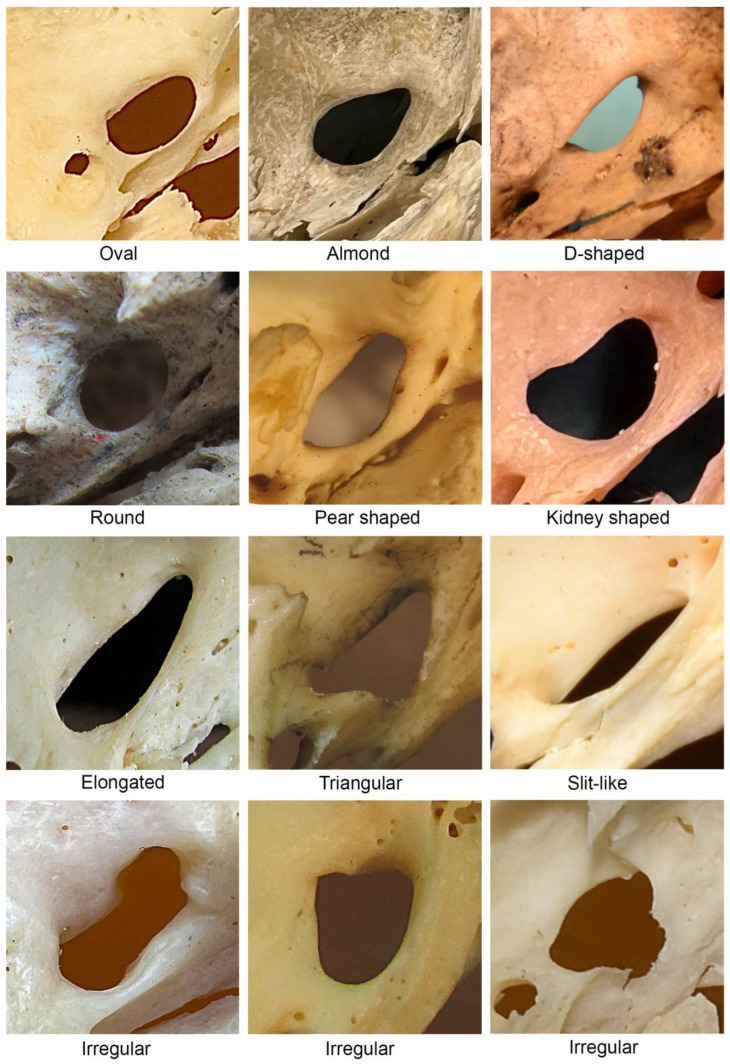
Shapes of the foramen ovale. Images were captured from the external aspect of the cranial base. The upper part of the image corresponds to the anterior, the right part to the medial, the left part to lateral, and the bottom part to the posterior aspect of the cranial base.

The most frequently observed shape of the FO was oval (37.1%), followed by almond (28.1%), irregular (21.0%), D-shaped (4.5%), round (3.0%), pear (1.9%), kidney (1.5%), elongated (1.5%), triangular (0.7%), and slit-like (0.7%) shape. The different FO shapes noted in the present study are shown in Figure 1, while the classification and distribution of FO shapes are summarized in Table 2. There were no statistically significant differences between the left and right sides.

Irregular shapes of the FO were a result of marginal bony outgrowths, confluence with other foramina, and complete (5.6%) or an incomplete (8.2%) pterygospinous bar, present either unilaterally or bilaterally.

**Figure 2:**
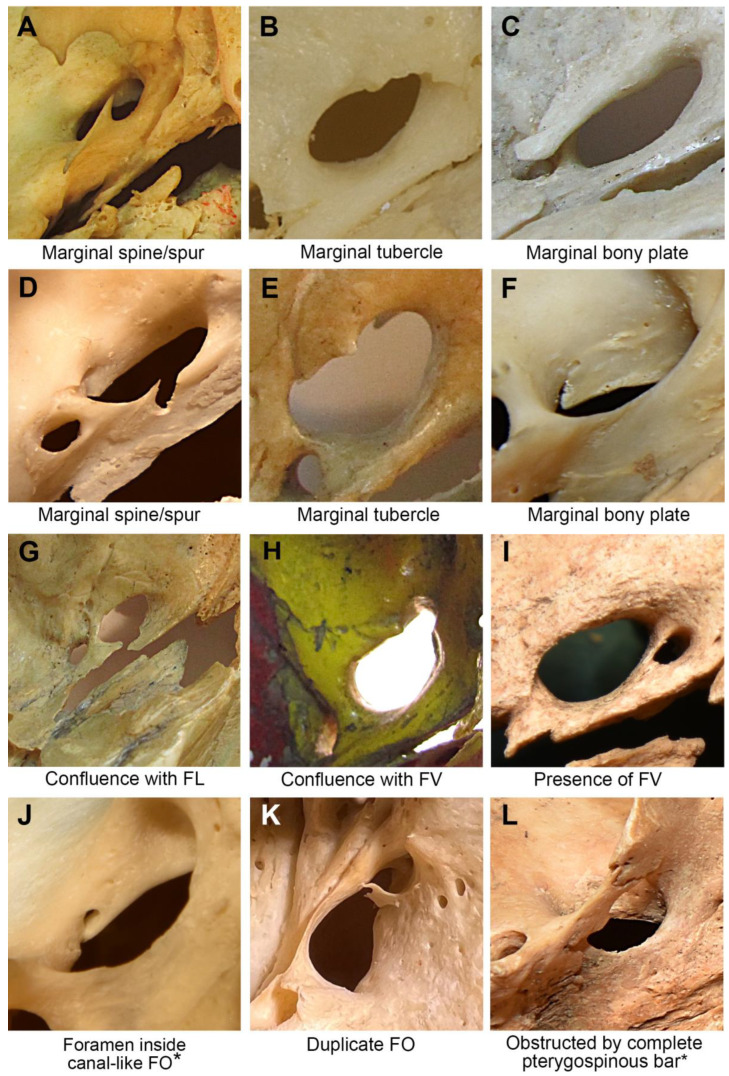
Foramen ovale (FO) with aberrant anatomical configurations. * Images were captured from lateral to medial direction on the external aspect of the cranial base. Images (**B**,**C**,**E**,**G**,**H**,**I**) were captured from the internal aspect of the cranial base. Images (**A**,**D**,**F**,**J**,**K**,**L**) were captured from the external aspect of the cranial base. FV—foramen of Vesalius. FL—foramen lacerum.

Marginal bony outgrowths were observed in 45 of the 267 (16.6%) skull halves: spines in 24 (9.0%), bony plates in 13 (4.9%), and tubercles in 8 (3.0%). A total of 12 (4.5%) foramina exhibited an irregular marginal morphology as a result of the presence of small marginal outgrowths that did not conform to any of the previously reported classifications. A small foramen was present inside the canal-like FO in 3 cases out of 267 (1.1%). Aberrant anatomical configurations of FO are depicted in Figure 2.

The confluence of the FO and the foramen lacerum (FL) was observed in 17 (6.4%) skull sides, 7 (2.6%) unilaterally, and 5 (3.8%) bilaterally. The confluence of the FO with an accessory foramen was observed in 3 (1.1%) skull sides, while the confluence of the FO and foramen of Vesalius was observed in 1 skull side (0.4%). One duplication (0.4%) of the FO due to a bony plate was noted (Figure 2).

Additionally, the analyzed FO were classified as either foramen-like (62.2%) or canal-like (37.8%). The incidence of a foramen-like FO was higher on both sides, 65.7% on the right and 58.5% on the left side.

## 4. Discussion

The results of the morphometric analysis of the 267 FO were consistent with those reported in other studies conducted on populations of European, American, African, and Asian descent [3,4,5,6,8,10,12,16,17,18,19,20,21,22,23,24,25,26,27,28,29,30,31]. However, the majority of existing morphometric studies of FO were limited to measurements of FO length and width (as presented in Table 3).

In the present study, the shortest width of an FO measured was 1.30 mm on the right side and 2.33 mm on the left side. It has been suggested that the presence of a narrow FO may result in a restriction of blood flow and possible ischemia of the trigeminal ganglion [7]. Alterations in blood flow and variations in the shape of the venous plexus inside the foramen can affect the mandibular branch of the trigeminal nerve and might therefore be another potential mechanism of trigeminal neuralgia [32]. Li et al. inferred that a narrow FO is associated with primary trigeminal neuralgia and its recurrence after microvascular decompression [33]. Furthermore, a small transverse diameter of an FO may affect the feasibility and safety of transoval cannulation during diagnostic and therapeutic procedures and consequently contribute to adverse events, including blindness, brainstem hematoma, temporal hematoma, carotid artery hemorrhage, and death [34,35,36]. A reduced size of an FO may be seen in patients with Paget’s disease or osteopetrosis due to structural deformity of the skull base [37]. In contrast, in case of an abnormally enlarged FO, neurinoma of the trigeminal nerve and parasellar tumors should be considered in the differential diagnosis [38].

**Table 3:** Comparison of FO dimensions between the present and previous studies.

Authors. Year (Country)	Number of Skull Sides	Longest Axis of FO (mm)		Shortest Axis of FO (mm)	
		Right Side Mean ± SD	Left Side Mean ± SD	Right Side Mean ± SD	Left Side Mean ± SD
Berlis et al., 1992 (Germany) [31]	120	7.41 ± 1.31		3.91 ± 0.77	
Ray et al., 2005 (Nepal) [17]	70	7.46 ± 1.41	7.01 ± 1.41	3.21 ± 1.02	3.29 ± 0.85
Osunwoke et al., 2010 (Nigeria) [18]	174	7.01 ± 0.1	6.98 ± 0.09	3.37 ± 0.07	3.33 ± 0.07
Somesh et al., 2011 (India) [12]	164	7.64 ± 1.19	7.56 ± 1.12	5.13 ± 0.83	5.24 ± 0.95
Desai et al., 2012 (India) [27]	250	8.14 ± 1.42	7.98 ± 1.89	5.26 ± 0.93	5.88 ± 1.01
Patil et al., 2013 (India) [16]	104	7.00 ± 2.17	6.80 ± 1.40	5.00 ± 0.42	4.70 ± 0.91
Gupta and Rai. 2013 (India) [6]	70	7.23 ± 1.14	6.49 ± 1.31	3.57 ± 0.70	3.50 ± 0.75
Unver Dogan et al., 2014 (Turkey) [29]	62	7.18 ± 1.78	7.29 ± 0.94	4.32 ± 1.41	4.06 ± 0.66
Murugan and Saheb. 2014 (India) [39]	500	8.9 ± 1.7	8.5 ± 1.3	3.7 ± 1.0	3.9 ± 1.0
Srimani et al., 2014 (India) [23]	80	7.75 ± 1.16	7.70 ± 1.14	3.41 ± 0.70	3.56 ± 0.89
Ashwini et al., 2017 (India) [24]	110	6.59 ± 2.21	6.38 ± 2.52	4.83 ± 0.97	4.59 ± 0.97
Bokhari et al., 2017 (Pakistan) [28]	110	7.04 ± 1.08	7.18 ± 1.14	5.15 ± 0.92	3.99 ± 0.86
Natsis et al., 2017 (Greece) [8]	195	7.63 ± 1.17	7.48 ± 1.20	4.47 ± 1.00	4.59 ± 1.00
Poornima et al., 2017 (India) [40]	200	6.5 ± 1.4	6.4 ± 1.5	3.54 ± 0.57	3.73 ± 0.83
Rao et al., 2017 (India) [41]	100	7.24 ± 0.89	7.11 ± 1.00	3.75 ± 0.71	3.75 ± 0.67
Srikantaiah et al., 2017 (India) [42]	80	7.45 ± 3.1	6.8 ± 1.5	6.0 ± 1.7	5.6 ± 1.4
Zdilla et al., 2017 (USA) [43]	169	6.62 ± 1.12	5.99 ± 1.08	3.13 ± 0.66	3.02 ± 0.63
Sophia et al., 2018 (India) [44]	222	7.57 ± 1.55	7.39 ± 1.53	4.3 ± 0.9	4.6 ± 1.1
Sankaran et al., 2018 (India) [22]	128	7.45 ± 1.1	7.61 ± 1.15	3.99 ± 1.8	4.6 ± 1.4
Prakash et al., 2019 (India) [19]	124	7.74 ± 1.94	7.60 ± 1.25	5.18 ± 0.98	5.4 ± 0.85
Das et al., 2019 (India) [25]	100	7.11 ± 1.69	6.53 ± 1.33	3.15 ± 0.69	3.20 ± 0.68
Kirwale and Sukre, 2020 (India) [45]	224	7.52 ± 1.15	7.29 ± 1.15	4.18 ± 0.78	4.28 ± 0.81
Akcay et al., 2021 (Turkey) [30]	80	7.09 ± 1.07	7.06 ± 1.01	4.16 ± 0.79	4.15 ± 0.5
Jyothi Lakshmi and Asharani, 2021 (India) [20]	110	8.4 ± 1.6	8.5 ± 1.3	4.5 ± 0.8	4.1 ± 0.6
Kastamoni et al., 2021 (Turkey) [21]	316	6.05 ± 1.01	5.86 ± 0.92	3.35 ± 0.83	3.37 ± 0.75
Açıkgöz et al., 2022 (Turkey) [46]	70	6.29 ± 0.15	6.00 ± 0.16	2.94 ± 0.10	2.83 ± 0.09
Hereus et al., 2022 (Belgium) [26]	118	7.41 ± 1.30	7.57 ± 1.07	4.63 ± 0.86	4.33 ± 0.99
Kaur et al., 2022 (India) [10]	200	8.16 ± 1.56	7.68 ± 1.25	4.97 ± 1.16	4.74 ± 1.21
Present study. 2023 (Slovenia)	267	7.13 ± 1.34	7.20 ± 1.29	3.71 ± 0.81	3.88 ± 0.84

FO—foramen ovale. SD—standard deviation.

This study noted significant variability in the shape of the FO; however, no statistically significant differences were observed between the left and right sides. The most commonly observed shape was oval, followed by almond, irregular, D-shaped, round, pear, kidney-shaped (also described as crescent or semilunar [11,23]), elongated, triangular, and slit-like (as shown in Figure 1). Previous studies also reported substantial variability in the distribution of different FO shapes, with no significant differences noted between sides (as presented in Table 4).

Variations in the shapes of FO should be considered a potential contributing factor to the failure of transoval access. An altered FO shape may indicate nasopharyngeal carcinoma, which tends to invade the intracranial space through the foramen [47].

The variability in size and shape of FO across different world regions has been explained by population variation, as well as embryologically since the sphenoid bone develops from both intramembranous and endochondral ossification [4,9,50,51]. During fetal development, the mandibular nerve migrates to its final position within the FO and is surrounded by a membranous bone. The first center of ossification in this region appears during the eighth week of fetal development, and the earliest formation of a fully formed ring-shaped FO is observed during the seventh month of fetal life. Overossification during the developmental process of the sphenoid bone comprising the FO may, however, result in morphologic abnormalities, such as spines, tubercles, bony bars, plates, or foramina, which may compress the mandibular nerve, causing trigeminal neuralgia. In addition, they may seriously hinder diagnostic and therapeutic procedures through the FO [5,50,52,53].

The present study observed marginal bony outgrowths of FO in 45 out of 267 (16.6%) analyzed skull sides. Similar findings were reported by Das et al. [25], Berlis et al. [31], and Gupta et al. [6]. The incidence of marginal projections reported by other authors varied from roughly 7% to as much as 24% [10,11,12,14,15,17,24,54]. Kastamoni et al. reported only 2 cases (1.1%) of bony protrusion into the FO [21]. We observed 24 spines (9.0%), 13 bony plates (4.9%), and 8 tubercles (3.0%) in 267 skull sides. Additional 12 (4.5%) foramina exhibited irregular marginal morphology due to small outgrowths that did not conform to previously reported classifications. Marginal irregularities were determined to be non-post-mortem, as the edges were smooth. These findings are consistent with those reported in previous studies [2,31].

In the present study, one duplication of the FO was observed (Figure 2). The unusual position or absence of a typical FO may manipulate the anatomical organization of neurovascular structures passing through the foramen. This may result in a lateral disposition of the mandibular nerve and entrapment of its branches between the bone and the neighboring muscles, causing trigeminal neuralgia [55].

The presence of a pterygospinous bar may reduce the space between the lateral pterygoid plate and the spine of the sphenoid bone and consequently preclude the cannulation of the FO [56]. When encountering difficulties accessing the FO with the needle despite attempting various angles, it is important for the surgeon to consider the potential presence of a pterygospinous bar. In such cases, intraoperative CT-guided neuronavigation can be utilized to successfully navigate the needle and increase the safety of the surgical procedure [57]. In the present study, 15 complete (5.6%) and 22 incomplete (8.2%) pterygospinous bars were observed.

Cannulation of the FO is utilized in the percutaneous treatment of trigeminal neuralgia and biopsy of lesions in the cavernous sinus [58] or deep lesions that otherwise require open surgical biopsy or craniotomy, namely, squamous cell carcinoma, meningioma, Meckel cave lesions [59,60], and electroencephalographic analysis of temporal seizures in patients undergoing selective amygdalohippocampectomy [61]. The shape and dimensions of the foramen may therefore be important in determining the appropriate caliber of a stylet that could be transmitted through the FO [62].

The FO serves as a landmark for percutaneous trigeminal rhizotomy in patients with trigeminal neuralgia (TN). The FO puncture is followed by destruction of TN fibers using radiofrequency thermocoagulation, balloon compression, or glycerol rhizotomy [63,64,65,66,67]. During cannulation, a misplaced needle in the foramen of Vesalius (FV) can cause severe complications, such as intracranial bleeding [68], as the distance between these two foramina is relatively short, between 0.93 and 5.45 mm [69]. In the present study, the mean distance between the FO and the FV was 4.26 mm on the right side and 2.52 mm on the left side. The minimal distance was 1.16 mm on the right and 0.83 mm on the left side.

The failure of percutaneous approaches may also be attributed to the misidentification of a large FV as the FO on imaging [70]. In the present study, the maximum diameter of the FV was 3.25 mm on the right side and 3.05 mm on the left side.

The middle meningeal vessels and the meningeal branch of the mandibular nerve may also sustain injuries during rhizotomy since the foramen spinosum is located very close to the FO [15,71,72,73,74]. In the present study, the mean distance between the FO and the FS was 3.04 ± 1.31 mm on the right side and 3.01 ± 1.11 mm on the left side. The shortest distance between the foramina was 0.25 mm on the right side and 0.72 mm on the left side.

The analyzed FO were additionally classified as either foramen-like or canal-like, as previously proposed by Elnashar et al. to highlight the correlation between the anatomical shapes of FO and the surgical view. A canal-like FO may hinder access to the middle cranial fossa [11].

Our study has a few limitations. First, we could not identify the sex and age of individuals from whom the skulls were obtained and consequently could not characterize the anthropometric evaluations based on these parameters. Second, the exact cause of variations observed in the present study is difficult to determine, although, in general, we consider that these may be due to genetic, nutritional, or environmental factors. However, because we had no autopsy data, it was impossible to exclude any potential underlying disease that would cause pathologic changes in the size, shape, or spatial disposition of the skull foramina. Finally, despite the meticulous precautions taken in the study protocols to minimize individual errors and subjectivity, we cannot absolutely exclude potential bias in evaluations.

## 5. Conclusions

A thorough understanding of the anatomy of the FO and its variations is essential in a number of diagnostic and therapeutic neurosurgical and anesthetic procedures. In this study, we report morphologic and morphometric characteristics of the FO in skulls from the Slovenian population and highlight the clinical relevance of the anatomical features. Our findings indicate a substantial degree of interindividual variability in the shape, size, and aberrant anatomical relationships of the FO, which has the potential to impact the feasibility and safety of relevant procedures.

## Figures and Tables

**Table 1:** Morphometric data on the foramen ovale.

Parameter	Mean ± SD (mm)		Range (mm)	
	Right Side	Left Side	Right Side	Left Side
	*n* = 138	*n* = 128	*n* = 138	*n* = 128
Length of FO	7.13 ± 1.34	7.20 ± 1.29	4.19–10.55	4.72–11.81
Width of FO	3.71 ± 0.81	3.88 ± 0.84	1.30–7.22	2.33–6.43
Distance between FO and FS	3.04 ± 1.31	3.01 ± 1.11	0.25–7.72	0.72–5.98
Distance between FO and FV	4.26 ± 2.85	2.52 ± 1.34	1.16–9.44	0.83–5.33

FO—foramen ovale. FS—foramen spinosum. FV—foramen of Vesalius. SD—standard deviation.

**Table 2:** Distribution of foramen ovale shapes.

Shape of FO	Oval (%)	Almond (%)	D-Shaped (%)	Round (%)	Pear (%)	Kidney (%)	Elongated (%)	Triangular (%)	Slit (%)	Irregular (%)
Right side (*n* = 137)	38.7	27.7	4.4	2.2	2.9	1.5	1.5	1.5	0.7	19.0
Left side (*n* = 130)	35.4	28.5	4.6	3.8	0.8	1.5	1.5	0	0.8	23.1
Overall proportion (*n* = 267)	37.1	28.1	4.5	3.0	1.9	1.5	1.5	0.7	0.7	21.0

FO—foramen ovale.

**Table 4:** Comparison of FO shapes between the present and previous studies.

Authors. Year (Country)	Number of Skull Sides	Oval (%)		Almond (%)		Irregular (%)		D-Shaped (%)		Round (%)		Pear (%)		Kidney (%)		Elongated (%)		Triangular (%)		Slit-Like (%)	
		R	L	R	L	R	L	R	L	R	L	R	L	R	L	R	L	R	L	R	L
Ray et al., 2005 (Nepal) [17]	70	62.8	60.0	31.4	37.1	/		/		2.8	2.8	/		/		/		/		1.14	
Somesh et al., 2011 (India) [12]	164	58.53	54.87	29.26	28.04	2.43	4.87	/		9.75	12.19	/		/		/		/		/	
Daimi et al., 2011 (India) [13]	180	29.87		/		/		46.16		12.52		/		/		10.41		/		1.04	
Desai et al., 2012 (India) [27]	250	62.8		23.2		/		/		11.81		/		/		/		/		/	
Wadhwa et al., 2012 (India) [48]	60	63.33	76.67	20.00	10.00	/		/		10.00	10.00	/		/		/		/		6.67	3.33
Gupta and Rai, 2013 (India) [6]	70	57.14	51.43	40.00	31.43	/		/		2.86	14.29	/		/		/		/		0.00	2.86
Murugan and Saheb, 2014 (India) [39]	500	69		29		0		/		2		/		/		/		/		/	
Patel and Mehta, 2014 (India) [49]	200	64	55	12	12	/		/		23	32	/		/		/		/		1	1
Srimani et al., 2014 (India) [23]	80	67.5	60	22.5	20	5	7.5	/		2.5	5	/		2.5	2.5	/		/		/	
Ashwini et al., 2017 (India) [24]	110	69.09	63.63	9.09	16.36	14.50	18.18	/		7.27	1.81	/		/		/		/		/	
Bokhari et al., 2017 (Pakistan) [28]	110	72.7	74.5	5.4	3.6	0	2.8	/		16.3	12.7	/		/		/		3.6	5.4	1.8	0
Sophia et al., 2018 (India) [44]	222	68.46		3.15				15.31		8.55		/		/		/		/		0.9	
Natsis et al., 2018 (Greece) [8]	195	49.6	62.6	23.5	14.8	19.1	13.9	/		7.8	8.7	/		/		/		/		/	
Das et al., 2019 (India) [25]	100	32	38	10	8	/		/		4	4	/		/		/		2	2	/	
Prakash et al., 2019 (India) [19]	124	64.5	56.4	25.8	30.6	1.62	4.8	/		8.0	8.0	/		/		/		/		/	
Akcay et al., 2021 (Turkey) [30]	80	70	70	17.5	20	/		/		5	5	/		/		/		/		7.5	5
Jyothi Lakshmi et al., 2021 (India) [20]	110	67.3	70.9	10.9	14.5	/		/		21.8	14.5	/		/		/		/		/	
Kastamoni et al., 2021 (Turkey) [21]	316	81.0		14.9		/		/		7.0		/		1.9		/		/		/	
Raguž et al., 2021 (Croatia) [9]	78	41.0	71.8	7.7	7.7	/		/		48.7	17.9	/		/		2.6	2.6	/		/	
Açıkgöz et al., 2022 (Turkey) [46]	70	34.29		34.29		/		10		12.85		/		/		/		/		8.57	
Kaur et al., 2022 (India) [10]	200	68	72	20	18	/		6	6	4	2	0	1	/		/		1	0	1	1
Santhosh et al., 2022 (India) [14]	102	43.1	52.9	19.6	19.6	0	3.9	11.8	9.8	7.8	2	/		/		9.8	7.8	/		7.8	3.9
Present study. 2023 (Slovenia)	267	38.7	35.4	27.7	28.5	19.0	23.1	4.4	4.6	2.2	3.8	2.9	0.8	1.5	1.5	1.5	1.5	1.5	0	0.7	0.8

## Data Availability

Data from this study are available upon reasonable request.

## Abbreviations

FO: foramen ovale. FS: foramen spinosum. FV: foramen of Vesalius. FL: foramen lacerum. R: right. L: left. SD: standard deviation.
